# Epidemiological characteristics and risk factors of lung cancer in a population with incidental pulmonary nodules in China: a prospective multicenter study of 10,560 cases

**DOI:** 10.1016/j.jncc.2026.03.003

**Published:** 2026-04-16

**Authors:** Wenhua Liang, Yongzhao Zhou, Chengping Hu, Wei Xiao, Jian Zhang, Min Li, Wei Wang, Xianwei Ye, Xiangyan Zhang, Ning Chang, Chunmei Yun, Dejun Sun, Yunhui Zhang, Zheng Qin, Wei Wang, Yong Zhang, Xin Zhang, Yi Xiang, Jieming Qu, Wei Zhang, Longhua Sun, Yang Hu, Jinfu Xu, Di Wu, Jianping Zhao, Xiaoju Zhang, Hairong Bao, Xiaoju Liu, Yubiao Guo, Zhanqing Li, Qichuan Zhang, Weiyi Zhu, Lan Yang, Xiaomin Dang, Baohui Han, Zhaohui Tong, Feng Wang, Liping Liu, Minhua Peng, Shuangxiu Wu, Liuhong Zeng, Lin Teng, Xiangcheng Qiu, Bo Wang, Jinsheng Tao, Meng Yang, Junjie Zhao, Silvia S. Chen, Jianbing Fan, Weimin Li, Dan Liu, Jianxing He, Nanshan Zhong

**Affiliations:** a Department of Thoracic Surgery/Oncology, The First Affiliated Hospital of Guangzhou Medical University, Guangzhou Institute of Respiratory Disease & Health, China State Key Laboratory and National Clinical Research Center for Respiratory Disease, Guangzhou, China; b Department of Respiratory Medicine, West China Hospital of Sichuan University, Chengdu, China; c Department of Respiratory Medicine, Xiangya Cancer Center, Xiangya Hospital, Central South University, Changsha, China; d Department of Respiratory Medicine, QILU Hospital, Shandong University, Jinan, China; e Department of Pulmonary Medicine, Xijing Hospital, Air Force Medical University of PLA, Xi’an, China; f Department of Respiratory and Critical Care Medicine, Guizhou Provincial People’s Hospital, Guiyang, China; g NHC Key Laboratory of Diagnosis & Treatment of COPD, Inner Mongolia Key Laboratory of Respiratory Diseases, Inner Mongolia People’s Hospital, Hohhot, China; h Department of Respiratory Medicine, The First People’s Hospital of Yunnan Province, Kunming, China; i Department of Respiratory Medicine, The First Hospital of China Medical University, Shenyang, China; j Department of Pulmonary Medicine, Zhongshan Hospital, Fudan University, Shanghai, China; k Department of Pulmonary & Critical Care Medicine, Ruijin Hospital, Shanghai Jiao Tong University School of Medicine, Shanghai, China; l Department of Respiration, The First Affiliated Hospital of Nanchang University, Nanchang, China; m Department of Respiratory Medicine, Shanghai Pulmonary Hospital, Tongji University School of Medicine, Shanghai, China; n Department of Pulmonary and Critical Care Medicine, Shenzhen Institute of Respiratory Diseases, The Second Clinical Medical College, Jinan University (Shenzhen People’s Hospital), The First Affiliated Hospital of Southern University of Science and Technology (Shenzhen People’s Hospital), China; o Department of Respiratory and Critical Care Medicine, Tongji Hospital, Tongji Medical College, Huazhong University of Science and Technology, Wuhan, China; p Department of Respiratory Medicine, Henan Provincial People’s Hospital, Zhengzhou, China; q Department of Gerontol Respiratory Medicine, The Frist Hospital of Lanzhou University, Lanzhou, China; r Department of Pulmonary Medicine, The First Affiliated Hospital of Sun Yat Sen University, Guangzhou, China; s Department of Cardiothoracic Surgery, The Second Affiliated Hospital of Xiamen Medical College, Xiamen, China; t Department of Respiratory and Critical Care Medicine, Shantou Central Hospital, Shantou, China; u Department of Pulmonary and Critical Care Medicine, The First Affiliated Hospital, Xi'an Jiaotong University, Xi’an, China; v Department of Pulmonary Medicine, Shanghai Chest Hospital, Shanghai Jiaotong University, Shanghai, China; w Department of Respiratory and Critical Care Medicine, Beijing Institute of Respiratory Medicine, Beijing Chaoyang Hospital, Capital Medical University, Beijing, China; x Department of Clinical Laboratory, State Key Laboratory of Respiratory Disease and National Clinical Research Centre for Respiratory Disease, The First Affiliated Hospital of Guangzhou Medical University, Guangzhou Medical University, Guangzhou, China; y AnchorDx Medical Co., Ltd., Guangzhou, China; z Interventional Oncology at Johnson and Johnson, New Brunswick, United States; aa Department of Pathology, School of Basic Medical Science, Southern Medical University, Guangzhou, China; bb National Clinical Research Center for Respiratory Disease, State Key Laboratory of Respiratory Diseases, The First Affiliated Hospital of Guangzhou Medical University, Guangzhou, China

**Keywords:** Pulmonary nodules, Epidemiological characteristics, Risk factors, Lung cancer

## Abstract

**Background:**

Lung cancer incidence and mortality continue to rise in China, highlighting the need for a deeper understanding of its epidemiology and optimal screening targets.

**Methods:**

A total of 10,560 participants with 5–30 mm pulmonary nodules incidentally detected via low-dose computed tomography (LDCT)/CT were enrolled from 23 hospitals (October 2018–May 2021) and followed for 2–3 years for evaluation of circulating tumor DNA (ctDNA) biomarkers to distinguish malignant and benign nodules. Demographic, clinical, and imaging data were collected and analyzed. Lung cancer risk factors were assessed using stepwise single and multiple-factor binary logistic regression. This study reports only the baseline epidemiological characteristics of the population and the associated risk factors of lung cancer.

**Results:**

A higher proportion of females (56.56%) were present across all age groups, with a median age of 53 years (45–62), younger than males (55 years [46–63], *P* < 0.001). While 97.1% of females and 37.8% of males were non-smokers, and 68.65% of participants had no occupational exposure, 71.10% had a history of second-hand smoke exposure. Most nodules were solitary (81.24%), sub-centimeter (78.22%), and non-solid (62.49%). Malignancy was identified in 17.33% of nodules, with 97.32% classified as early-stage lung adenocarcinoma (AJCC stage 0–I: 90.35%). The malignant rate increased with age and nodule size and was also associated with multiple nodules, ground-glass nodules (GGNs), upper lobe location, and female sex. Proportion of patients with malignant nodules was similar between non-high-risk (21.04%) and high-risk (17.92%) groups based on current Chinese screening guidelines, while proportions of benign nodules were notably high for solid nodules (37.21%) and small nodules (20.15%).

**Conclusion:**

Our large-scale investigation provides updated data to guide future precise clinical management of pulmonary nodules, with a particular focus on younger females and reducing overtreatment.

## 1 Introduction

Lung cancer is the second most common cancer with 2.2 million new cases and 1.8 million deaths worldwide in 2020.^1^ Because lung cancer is often diagnosed at a late stage, the 5-year survival rate is below 20% in most countries. On the contrary, early-stage lung cancers hold over 80% 5-year survival rates.^2^ The use of chest low-dose computed tomography (LDCT) for lung cancer screening changed status of lung cancer in the past 20 years.^3^ Several large-scale randomized controlled trials have demonstrated a significant reduction in lung cancer mortality through either annual LDCT screening or volume CT screening for high-risk populations,^4^^,^^5^ such as a 20% reduction in the NLST study^4^ and a 24%−33% reduction in the NELSON study.^5^ Therefore, early detection and appropriate treatment can significantly improve the life expectancy of lung cancer patients.

In China, the incidence and mortality rates of lung cancer have gradually risen, making it the leading cause of cancer-related cases and deaths.^6^ Multiple large-scale community-based lung cancer screening programs have been implemented using LDCT. In Hebei Province from 2013 to 2019, 3697 lung nodule (12.73%) and 421 suspected lung cancer cases (1.46%) were detected in 28,899 high-risk participants. The majority of nodules were solid nodules (81.03%).^7^ In Shanghai, among 6717 participants enrolled between 2013 and 2014 and followed for three years, 804 lung nodules (22.9%) and 51 lung cancer cases (1.5%) were detected.^8^ Additionally, according to the Chinese Expert Consensus on low-dose spiral CT lung cancer screening in 2015, individuals at high risk are defined as those aged 50–75 years with any of the following risk factors: a history of smoking ≥ 20 pack-years, quitting smoking within the past 15 years, exposure to passive smoke or high-risk occupations (*e.g.*, asbestos, plating, uranium, radon), or a history of chronic obstructive pulmonary disease (COPD), diffuse pulmonary fibrosis, pulmonary tuberculosis, malignant tumors, or a personal or family history of lung cancer,^9^ and there are around 2% lung cancer detected in non-high-risk participants.^8^ Between 2006 and 2017, a health examination program at Western China Hospital involving 15,996 participants reported that lung cancer in patients falling outside of screening criteria accounted for 75.6% in the non-high-risk population.^10^ Therefore, it is crucial to assess the current characteristics of the pulmonary nodule population and establish updated screening guidelines for targeted groups.

Previously we had developed a blood-based diagnostic test using high-throughput targeted DNA methylation sequencing of circulating tumor DNA (ctDNA) to differentiate between benign and malignant pulmonary nodules.^11^^,^^12^ To evaluate the diagnostic assay performance in real-world settings, we initiated a prospective, multi-center, observational clinical trial (NCT03651986) in 2018. The trial successfully enrolled 10,560 participants with incidental 5–30 mm pulmonary nodules, from 23 hospitals across 16 provinces in China. These patients’ pulmonary nodules were incidentally detected, not limited to the high-risk population of lung cancer, and not limited to any specific departments in the hospitals. Moreover, this is a non-interventional study and the patients have no pressure to make decision to enroll the study when being recruited. Therefore, although this is not a rigorous sampling survey, it can reflect real-world proportions of demographic, clinical and CT imaging features in the cohort. Here, we present large population-based epidemiological characteristics of pulmonary nodules in China at the baseline and analyze the risk factors associated with lung cancer.

## 2 Materials and methods

### 2.1 Study design and participants

This multicenter, prospective, observational cohort study (NCT03651986) was approved by the Ethics Committee of 23 participating hospitals in China^13^ (Supplementary Table 1). Between October 2018 and May 2021, a total of 10,560 participants aged 18 years or older with solitary or multiple non-calcified pulmonary nodules - including solid nodules (SNs), part-solid ground-glass nodules (mGGNs), and pure ground-glass nodules (pGGNs) - were enrolled. Each participant completed a patient pulmonary history questionnaire, provided informed consent, and was followed for 2–3 years. The inclusion and exclusion criteria were described previously^13^ and are detailed in the Supplementary materials. Briefly, eligible participants met the following inclusion criteria: (I) Age ≥18 years, of either sex; (II) Presence of pulmonary nodules detected by standard-dose or low-dose CT screening, with (i) non-calcified nodules measuring 5 to 30 mm in diameter and (ii) inclusion of three nodule types—solid nodules, part-solid nodules (mixed ground-glass nodules), and pGGNs; (III) Patients with newly detected pulmonary nodules on baseline CT were initially diagnosed within 60 days prior to enrollment; (IV) Willingness to complete the subjects’ lung history questionnaire; (V) Agreement to a 2–3 year follow-up and cooperation with relevant imaging, serological, surgical, and other examinations; and (VI) Ability to fully understand the informed consent, agree to participate in the study and sign the informed consent. Exclusion criteria were defined as: (I) Pregnant or lactating females; (II) Patients underwent any diagnostic puncture therapy, such as percutaneous lung biopsy, transbronchial biopsy or surgery prior to enrollment; (III) Receive any blood transfusion therapy within 30 days prior to enrollment; (IV) Patients with cancer confirmed pathologically within 2 years prior to enrollment (except for nonmelanoma skin cancer); and (V) Inability to understand or obtain informed written consent.

### 2.2 Procedures

Individuals incidentally found to have pulmonary nodules with 5–30 mm in diameter by a chest LDCT/CT scan (with a 0.625 mm slice thickness) at the designated hospitals or during a physical examination prior to visiting the designated hospitals, were recommended by the principal investigators (PIs) to join the study. Demographic and clinical information of participants were self-reported via a questionnaire (summarized in Supplementary Table 2) and collected by the PIs at the enrollment. All chest CT images were prospectively collected either during initial nodule check or prior to surgery at each clinical site. The pulmonary nodule sizes and imaging features were assessed by the radiologists in the designated hospitals. The nodule’s size is measured based on its long-axis diameter (maximum diameter) of the entire lesion (*i.e.* overall diameter) on lung windows of CT scans. For the mGGN, its solid portion in the nodule was assessed concurrently on mediastinal windows. The PIs reviewed and documented the imaging features from the CT scans, with detailed information and imaging characteristics summarized in Supplementary Table 3.

This is an observational clinical study. The decision for a participant to proceed with surgical treatment or follow up was made by the project investigators in the designated hospitals according to clinical guidelines. Participants with low or medium risk of malignancy were followed up. Solid nodules were followed for 2 years, and mGGN and pGGN were followed for 3 years according to the “Chinese expert consensus for lung cancer screening (2018 edition)”.^14^

### 2.3 Outcomes

Enrolled patients identified as high risk for lung cancer by physicians, based on clinical experience, underwent surgical treatment either at enrollment or during follow-up. Malignant and benign nodules are defined by definitive pathological diagnostic results obtained at the participating institutions. Benign nodules were also defined as those that spontaneously shrank or disappeared over time. Both solid nodules remained stable over 2 years of follow-up and mGGN/pGGN remained stable over 3 years of follow-up are commonly considered benign.^14^ However, the nodules that have finished 2–3 years follow-up are not used for lung cancer risk factor analysis in this study. The pathological classification described in this study was based on the 2015 World Health Organization (WHO) histological classification of lung cancer,^15^ while lung cancer staging was determined according to the eighth edition of the International Association for the Study of Lung Cancer (IASLC) and the American Joint Committee on Cancer (AJCC) stage classification for non-small-cell lung cancer (NSCLC) .^16^

### 2.4 Statistical analysis

Sample size calculation of the trial was described previously^13^ and also provided in Supplementary materials. Continuous variables were presented as number of cases, median ([Q1, Q3]), and categorical variables were presented as sample size, frequency, or percentage. Pairwise deletion was applied to delete the missing data. The differences between benign and malignant nodules in continuous variable were analyzed by two-sided *P*-value of Wilcoxon Rank Sum Test based on *t* approximation, and categorical variables by Chi-square test or Fisher's precision probability test. The above statistical analysis was implemented by the "CBCgrps" (version 2.8.2) R package. Univariate correlation analysis of risk factors associated with malignant pulmonary nodules was conducted by single factor binary logistic regression (Supplementary Tables 6 and 8). Univariate factors with a *P*-value < 0.05 were selected for multiple-factor binary logistic regression analysis (Fig. 3). Both regression analyses were performed using R software version 4.0.0, with a *P*-value < 0.05 considered statistically significant. The Hosmer-Lemeshow test was conducted to evaluate the goodness-of-fit of above logistic regression models in Fig. 2, Fig. 3 and Supplementary Fig. 3, with a *P*-value > 0.05 indicating a good fit between predicted and observed values. Forest plots were generated using the 'forestplot' (version 2.0.1) R package.

## 3 Results

### 3.1 Age, gender and smoking distribution

**Fig. 1:**
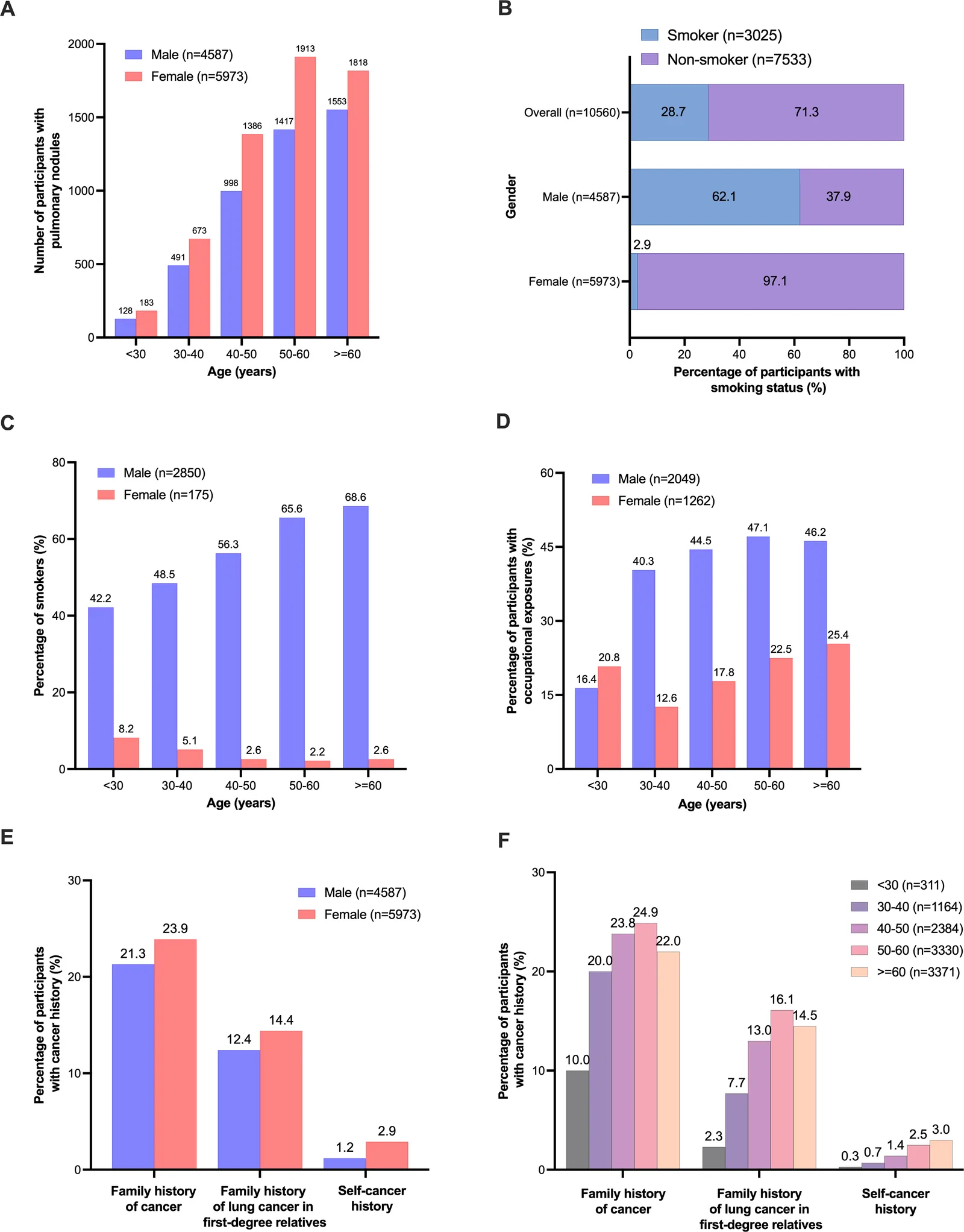
Epidemiological characteristics of the pulmonary-nodule population screened by low-dose computed tomography/computed tomography. (A) Population distribution in two genders. (B) Population distribution of smoking history in two genders. (C) Population distribution of smoking history in various ages. (D) Population distribution of occupational exposure history in various ages and genders. (E, F) Population distribution of family history of cancer or of lung cancer in first-degree relatives, and self-cancer history in two genders (E) and in various ages (F). The numbers in the brackets are the numbers of males, females and overall population in the cohort.

Females comprised the majority of the cohort (*n* = 5973, 56.56%) and were represented across all age groups. The median age for females was 53 (45–62) years, which was significantly younger than the 55 years median age of males (46–63) years (*P* < 0.001) (Fig. 1A). The number of females with nodules increased with age, reaching its peak at age 60 years, while the number of males continued to rise with age (Fig. 1A). A total of 71.34% (*n* = 7533/10,560) of participants had no smoking history in this cohort, of which 97.07% of females (*n* = 5798/5973) and 37.82% of males (*n* = 1735/4587) (Fig. 1B). In contrast, 94.21% (*n* = 2850/3025) of smokers were males, with smoking proportion increasing with age (Fig. 1C). Among the smokers, 44.03% (*n* = 1332/3025) were current smokers and 56.0% (*n* = 1694/3025) were former smokers (Supplementary Table 2). 71.10% (*n* = 7508/10,560) of participants had been exposed to second-hand smoke either at work or at home, with no statistically significant difference between females (70.73%, *n* = 3283/4587) and males (71.57%, *n* = 4225/5987) or across different age groups (Supplementary Fig. 1A). This suggests that, despite the majority of females being non-smokers, a significant number were still exposed to second-hand smoke.

About 31.35% (*n* = 3311/10,560) of participants had a history of occupational exposures, as shown in Table 1, which is similar to that (36.0–36.5%) in the high-risk populations of lung cancer reported by two large-scale population-based investigation in China.^17^^,^^18^ Notably, the rate of occupational exposure history was approximately 2.2 times higher in males than in females (45.05% *vs.* 20.84%, *P* < 0.001) and generally increased with age, except for females under 30 years old, who showed a higher rate of occupational exposure (20.82% *vs.* 16.43%) (Fig. 1D). Notably, about 78.53% of participants with an occupational exposure history also had a history of second-hand smoke exposure, while 34.63% of those with a second-hand smoke exposure history also had occupational exposure (Supplementary Fig. 1B).

**Table 1:** Demographic and clinical characteristics of overall participants^a^.

Characteristics	Total (*n* = 10,560)	At baseline		At follow up of 3 years^b^			Total benign (*n* = 623)	Total malignant (*n* = 2123)	*P* value^c^
		Pathologically benign (*n* = 298)	Pathologically malignant (*n* = 1394)	Benign - nodule disappear (*n* = 226)	Pathologically benign (*n* = 99)	Pathologically malignant (*n* = 729)			
Sample source, No. (%)									< 0.001
Inpatient	2072 (19.62)	224 (75.17)	1032 (74.03)	14 (6.19)	35 (35.35)	290 (39.78)	273 (43.82)	1322 (62.27)	
Outpatient	8483 (80.33)	74 (24.83)	362 (25.97)	212 (93.81)	64 (64.65)	439 (60.22)	350 (56.18)	801 (37.73)	
Age, median (Q1, Q3), years	54 (45, 62)	51 (45.25, 59)	55 (47, 63)	53 (43, 61)	53 (46, 60)	55 (47, 63)	52 (45, 60)	55 (47, 63)	< 0.001
Age classification, years									< 0.001
< 30	311 (2.95)	10 (3.36)	27 (1.94)	12 (5.31)	5 (5.05)	14 (1.92)	27 (4.33)^d^	41 (1.93)^d^	
30–40	1164 (11.02)	34 (11.41)	127 (9.11)	33 (14.60)	8 (8.08)	66 (9.05)	75 (12.04)^d^	193 (9.09)^d^	
40–50	2384 (22.58)	84 (28.19)	305 (21.88)	51 (22.57)	27 (27.27)	143 (19.62)	162 (26.00)^d^	448 (21.10)^d^	
50–60	3330 (31.53)	102 (34.23)	462 (33.14)	65 (28.76)	32 (32.32)	253 (34.71)	199 (31.94)	715 (33.68)	
≥ 60	3371 (31.92)	68 (22.82)	473 (33.93)	65 (28.76)	27 (27.27)	253 (34.71)	160 (25.68)^d^	726 (34.20)^d^	
Gender									< 0.001
Female	5973 (56.56)	145 (48.66)	876 (62.84)	98 (43.36)	51 (51.52)	486 (66.67)	294 (47.19)	1362 (64.15)	
Male	4587 (43.44)	153 (51.34)	518 (37.16)	128 (56.64)	48 (48.48)	243 (33.33)	329 (52.81)	761 (35.85)	
Clinical symptoms									< 0.001
No	7105 (67.28)	214 (71.81)	1057 (75.82)	132 (58.41)	65 (65.66)	548 (75.17)	411 (65.97)	1605 (75.60)	
Yes	3454 (32.71)	84 (28.19)	337 (24.18)	94 (41.59)	34 (34.34)	181 (24.83)	212 (34.03)	518 (24.40)	
Smoking history									< 0.001
No	7533 (71.34)	199 (66.78)	1074 (77.04)	135 (59.73)	63 (63.64)	583 (79.97)	397 (63.72)	1657 (78.05)	
Yes	3025 (28.65)	99 (33.22)	320 (22.96)	91 (40.27)	36 (36.36)	146 (20.03)	226 (36.28)	466 (21.95)	
Smoker gender									0.111
Female	175 (1.66)	1 (0.34)	15 (1.08)	2 (0.88)	1 (1.01)	6 (0.82)	4 (0.64)	21 (0.99)	
Male	2850 (26.99)	98 (32.89)	305 (21.88)	89 (39.38)	35 (35.35)	140 (19.20)	222 (35.63)	445 (20.96)	
NA	7535 (71.35)	199 (66.78)	1074 (77.04)	135 (59.73)	63 (63.64)	583 (79.97)	397 (63.72)	1657 (78.05)	
Nonsmoker gender									< 0.001
Female	5798 (54.91)	144 (48.32)	861 (61.76)	96 (42.48)	50 (50.51)	480 (65.84)	290 (46.55)	1341 (63.17)	
Male	1735 (16.43)	55 (18.46)	213 (15.28)	39 (17.26)	13 (13.13)	103 (14.13)	107 (17.17)	316 (14.88)	
NA	3027 (28.66)	99 (33.22)	320 (22.96)	91 (40.27)	36 (36.36)	146 (20.03)	226 (36.28)	466 (21.95)	
Secondhand smoke exposure									0.040
No	2764 (26.17)	63 (21.14)	385 (27.62)	54 (23.89)	37 (37.37)	235 (32.24)	154 (24.72)	620 (29.20)	
Yes	7508 (71.10)	231 (77.52)	982 (70.44)	163 (72.12)	59 (59.60)	481 (65.98)	453 (72.71)	1463 (68.91)	
NA	288 (2.73)	4 (1.34)	27 (1.94)	9 (3.98)	3 (3.03)	13 (1.78)	16 (2.57)	40 (1.88)	
Occupational exposure history									< 0.001
No	7227 (68.44)	207 (69.46)	1086 (77.91)	138 (61.06)	68 (68.69)	565 (77.50)	413 (66.29)	1651 (77.77)	
Yes	3311 (31.35)	90 (30.20)	302 (21.66)	88 (38.94)	31 (31.31)	164 (22.50)	209 (33.55)	466 (21.95)	
NA	22 (0.21)	1 (0.34)	6 (0.43)	0 (0)	0 (0)	0 (0)	1 (0.16)	6 (0.28)	
Cancer history									0.094
No	10,313 (97.66)	295 (98.99)	1371 (98.35)	225 (99.56)	98 (98.99)	712 (97.67)	618 (99.20)	2083 (98.12)	
Yes	226 (2.14)	2 (0.67)	18 (1.29)	1 (0.44)	1 (1.01)	17 (2.33)	4 (0.64)	35 (1.65)	
NA	21 (0.20)	1 (0.34)	5 (0.36)	0 (0)	0 (0)	0 (0)	1 (0.16)	5 (0.24)	
Family history of cancers									0.782
No	8156 (77.23)	242 (81.21)	1109 (79.56)	183 (80.97)	73 (73.74)	575 (78.88)	498 (79.94)	1684 (79.32)	
Yes	2400 (22.73)	56 (18.79)	285 (20.44)	43 (19.03)	26 (26.26)	154 (21.12)	125 (20.06)	439 (20.68)	
Lung cancer history^e^									0.241
No	8989 (85.12)	269 (90.27)	1208 (86.66)	197 (87.17)	77 (77.78)	604 (82.85)	543 (87.16)	1812 (85.35)	
Yes	1429 (13.53)	27 (9.06)	171 (12.27)	27 (11.95)	18 (18.18)	115 (15.78)	72 (11.56)	286 (13.47)	
NA	142 (1.34)	2 (0.67)	15 (1.08)	2 (0.88)	4 (4.04)	10 (1.37)	8 (1.28)	25 (1.18)	
BMI, median (Q1, Q3), Kg/m²	23.24 (21.23, 25.35)	23.51 (21.20, 25.40)	23.04 (20.96, 25.00)	23.71 (21.48, 25.95)	22.72 (20.86, 24.51)	23.23 (21.11, 25.39)	23.42 (21.26, 25.61)	23.05 (21.01, 25.15)	0.023
Education level									0.003
High	4439 (42.04)	95 (31.88)	612 (43.90)	101 (44.69)	37 (37.37)	316 (43.35)	233 (37.40)	928 (43.71)	
Low	5787 (54.80)	182 (61.07)	687 (49.28)	121 (53.54)	58 (58.59)	388 (53.22)	361 (57.95)	1075 (50.64)	
NA	334 (3.16)	21 (7.05)	95 (6.81)	4 (1.77)	4 (4.04)	25 (3.43)	29 (4.65)	120 (5.65)	
Nodule size long diameter, mm^f^									< 0.001
5 to 10	10,428 (78.22)	169 (46.43)	600 (40.19)	97 (66.90)	208 (79.39)	454 (55.57)	474 (61.48)	1054 (45.63)	
10 to 20	2377 (17.83)	147 (40.38)	636 (42.60)	40 (27.59)	49 (18.70)	304 (37.21)	236 (30.61)	940 (40.69)	
20 to 30	526 (3.95)	48 (13.19)	257 (17.21)	8 (5.52)	5 (1.91)	59 (7.22)	61 (7.91)	316 (13.68)	
Nodule type^f^									< 0.001
mGGN	3203 (24.03)	56 (15.38)	620 (41.53)	35 (24.14)	72 (27.48)	366 (44.80)	163 (21.14)	986 (42.68)	
pGGN	5127 (38.46)	84 (23.08)	502 (33.62)	49 (33.79)	83 (31.68)	340 (41.62)	216 (28.02)	842 (36.45)	
SN	4997 (37.48)	224 (61.54)	370 (24.78)	61 (42.07)	107 (40.84)	111 (13.59)	392 (50.84)	481 (20.82)	
NA	4 (0.03)	0 (0)	1 (0.07)	0 (0)	0 (0)	0 (0)	0 (0)	1 (0.04)	
Multi target nodule number									< 0.001
1	8579 (81.24)	286 (95.97)	1258 (90.24)	200 (88.50)	60 (60.61)	379 (51.99)	546 (87.64)	1637 (77.11)	
2	1476 (13.98)	12 (4.03)	120 (8.61)	20 (8.85)	32 (32.32)	241 (33.06)	64 (10.27)	361 (17.00)	
3	317 (3.00)	0 (0)	15 (1.08)	3 (1.33)	3 (3.03)	61 (8.37)	6 (0.96)	76 (3.58)	
4	114 (1.08)	0 (0)	0 (0)	2 (0.88)	4 (4.04)	28 (3.84)	6 (0.96)	28 (1.32)	
5	58 (0.55)	0 (0)	1 (0.07)	1 (0.44)	0 (0)	14 (1.92)	1 (0.16)	15 (0.71)	
6	11 (0.10)	0 (0)	0 (0)	0 (0)	0 (0)	4 (0.55)	0 (0)	4 (0.19)	
7	3 (0.03)	0 (0)	0 (0)	0 (0)	0 (0)	2 (0.27)	0 (0)	2 (0.09)	
8	2 (0.02)	0 (0)	0 (0)	0 (0)	0 (0)	0 (0)	0 (0)	0 (0)	
Pleural effusion									0.790
No	10,486 (99.30)	296 (99.33)	1385 (99.35)	223 (98.67)	99 (100.00)	723 (99.18)	618 (99.20)	2108 (99.29)	
Yes	74 (0.70)	2 (0.67)	9 (0.65)	3 (1.33)	0 (0)	6 (0.82)	5 (0.80)	15 (0.71)	
Lymph node metastasis									0.326
No	10,265 (97.21)	291 (97.65)	1318 (94.55)	220 (97.35)	96 (96.97)	706 (96.84)	607 (97.43)	2024 (95.34)	
Yes	60 (0.57)	2 (0.67)	16 (1.15)	1 (0.44)	0 (0)	5 (0.69)	3 (0.48)	21 (0.99)	
NA	235 (2.23)	5 (1.68)	60 (4.30)	5 (2.21)	3 (3.03)	18 (2.47)	13 (2.09)	78 (3.67)	

a Except for age and BMI expressed as median (Q1, Q3), other numbers outside the bracket refer to participant numbers. Numbers in the bracket refer to the percentage.

b The follow up period is from October 2018 to September 2023.

c *P* value is calculated by total malignant **vs.** total benign and lower than 0.05 means significance.

d *P* value lower than 0.05 on that age range.

e Lung cancer history refers to lung cancer history in immediate family member.

f The number is based on nodule numbers, not based on participant numbers. Abbreviations: BMI, body mass index; mGGN, mixed ground-glass nodule; mm, millimeter; NA, unknown; pGGN, pure ground-glass nodule; SN, solid nodule.

### 3.2 Family history of cancers

Compared to males, females had a higher rate of family history of cancers (23.85% *vs.* 21.26%, *P* = 0.002), a family history of lung cancer in first-degree relatives (14.40% *vs.* 12.40%, *P* = 0.003), and a personal history of cancer, with thyroid cancer being the most common (0.62% of total, Supplementary Table 2) (2.86% *vs.* 1.20%, *P* < 0.001, Fig. 1E). These findings suggest that the formation of pulmonary nodules in females may be linked to genetic factors. The proportion of participants with a family history of cancer gradually increased with age up to 60, after which it declined. In contrast, the proportion of participants with a personal history of cancer continued to rise across all age groups (Fig. 1F).

### 3.3 Clinical symptoms

Consistent with previous reports,^19^ the majority of participants (*n* = 7105, 67.28%) with pulmonary nodules were asymptomatic, while the remaining (*n* = 3454, 32.71%) had clinical symptoms. The most common symptom was coughing, 68.21% (*n* = 2356/3454) in all symptomatic cases. 46.78% (*n* = 4940) had comorbidities and 39.02% (*n* = 4121) had a history of disease or symptoms, as detailed in Supplementary Table 2.

### 3.4 Nodule size, location and morphology

A total of 13,331 target pulmonary nodules were identified in 10,560 participants, with 8579 participants (81.24%) having solitary nodules and 1981 (18.76%) harboring multiple nodules, where 13.98%, 3.00% and 1.78% of participants have two, three and more than three nodules, respectively. Notably, 78.22% (*n* = 10,428) nodules were at 5–10 mm in long diameter, 17.83% (*n* = 2377) at 10–20 mm, and only 3.95% (*n* = 526) at 20–30 mm (Table 1). For nodule location, majority of the nodules located at right upper (29.76% (*n* = 3967)) and left upper lobes (21.66% (*n* = 2887) of the lung, while 20.92% (*n* = 2789), 17.45% (*n* = 2326) and 9.95% (*n* = 1327) of the nodules located at the right lower, left lower, and middle lobe of the lung, respectively (Supplementary Table 3). Morphological characteristics of the nodules, including spiculation, lobulation, spinous, vacuole and eccentric thick-walled cavity (cancerous cavity) signs, as well as surrounding signs, were observed in <10.51% of the target nodules in the cohort (Supplementary Table 3). Additionally, 0.70% of participants had pleural effusion, and 0.57% had lymph node metastasis (Table 1).

### 3.5 Characteristics associated with GGN

**Fig. 2:**
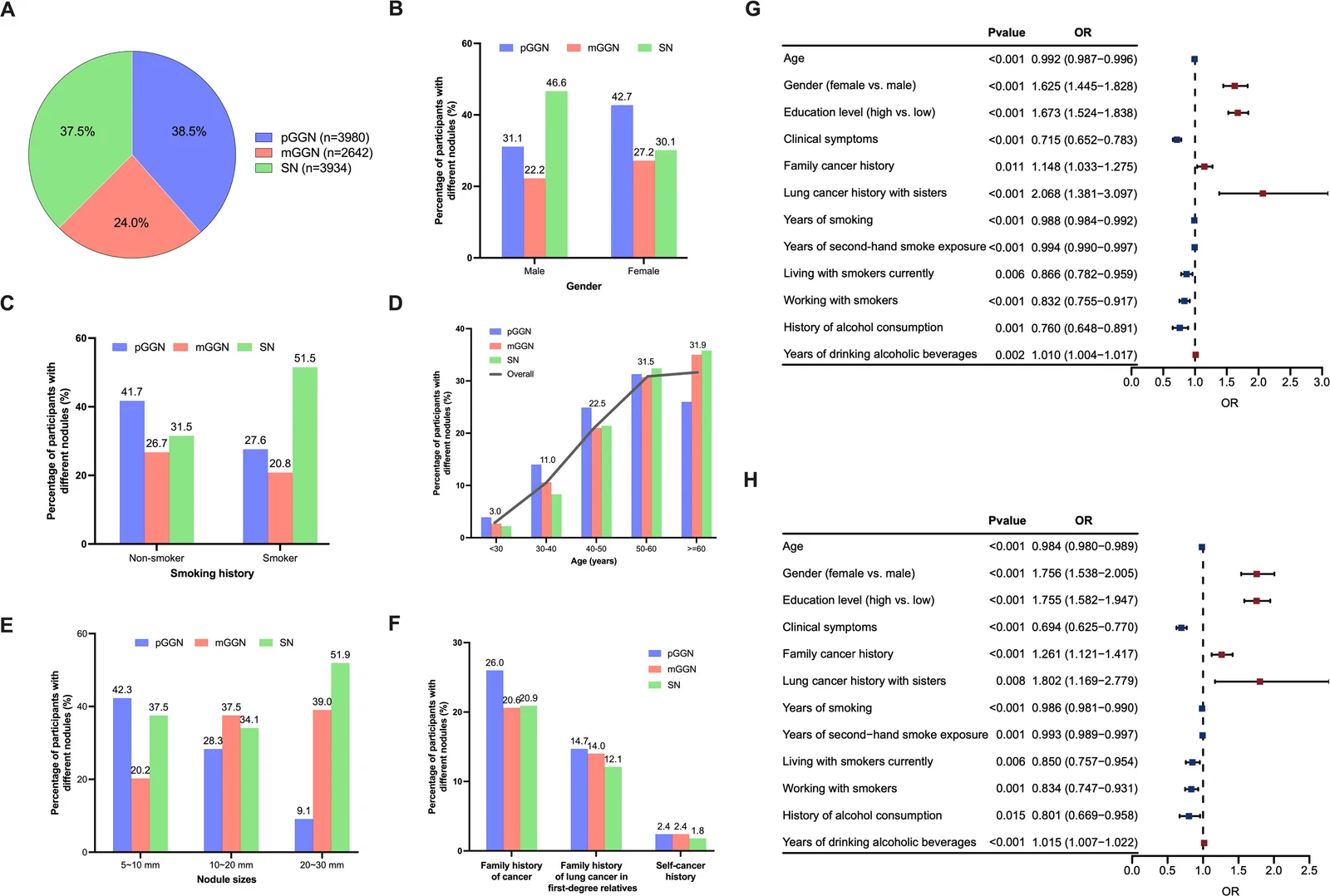
Epidemiological characteristics and risk factors of pGGNs, mGGNs and SNs. (A) Proportion of three types of nodules in the cohort. (B) Distributions by genders. (C) Distributions with smoking statues. (D) Distributions across ages. (E) Distributions in various nodule sizes. (F) Relationship with cancer histories. (G) Risk factors associated with pGGN and mGGN comparing with SN. (H) Risk factors associated with pGGN compared with SN. The risk factors shown in (G) and (H) are statistically significant in the stepwise backward multivariate analysis based on the univariates regression analysis as presented in Supplementary Table 5. All the factors significantly associated with malignant nodules in the univariate analysis were subjected to multivariate factor binary logistic regression analysis via R software, version 4.0.0. *P*-value lower than 0.05 is significant. The Hosmer-Lemeshow test was used to evaluate the goodness-of-fit of logistic regression models in Fig. 2G and H and shown in Supplementary Table 11. mGGN, mixed ground-glass nodule; OR, odds ratio; pGGN, pure ground-glass nodule; SN, solid nodule.

Based on the density of the largest pulmonary nodule in each participant, 38.46% (*n* = 3980) participants had pGGNs, 24.03% (*n* = 2642) had mGGNs, and 37.48% (*n* = 3934) had SNs (Fig. 2A and Table 1). A significant proportion of females (42.73%) and non-smokers (41.76%) had pGGNs, while SN was prevalent in males (46.62%) and smokers (51.54%) (Fig. 2B and C). Across all age ranges, the proportion of mGGN and SN increased with ages. However, pGGNs were more common in participants under 50 years old (17.86% for < 40 years and 24.85% for 40–50 years) while SN were more prevalent in those over 50 years old (32.36% for 50–60 years and 35.82% for ≥ 60 years) (Fig. 2D). pGGNs were also predominantly found in small nodules (42.30% at 5–10 mm and 28.31% at 10–20 mm), while higher percentage of mGGNs and SNs were more common in large nodules (39.0% and 51.9%, respectively in 20–30 mm nodules) (Fig. 2E).

Notably, pGGNs were more likely to occur in participants with a family history of cancer (*P* < 0.001) and lung cancer in first-degree relatives (*P* = 0.004), and the proportion of such family histories was slightly higher in the pGGN group compared to the mGGN and SN groups; in contrast, no significant association was found with a personal history of cancer (*P* = 0.142) (Fig. 2F and Supplementary Table 4). We also compared the epidemiological characteristics of the total GGNs (*i.e.* pGGN and mGGN) *vs.* SN and only pGGN *vs.* SN. And the results indicated that the pGGNs were closely associated with family history of lung cancer in first-degree relatives, particularly in sisters (Fig. 2G and H, Supplementary Table 5 and 11), highlighting the unique genetic background related to the occurrence of GGNs.

### 3.6 Risk factors associated with malignancy

**Fig. 3:**
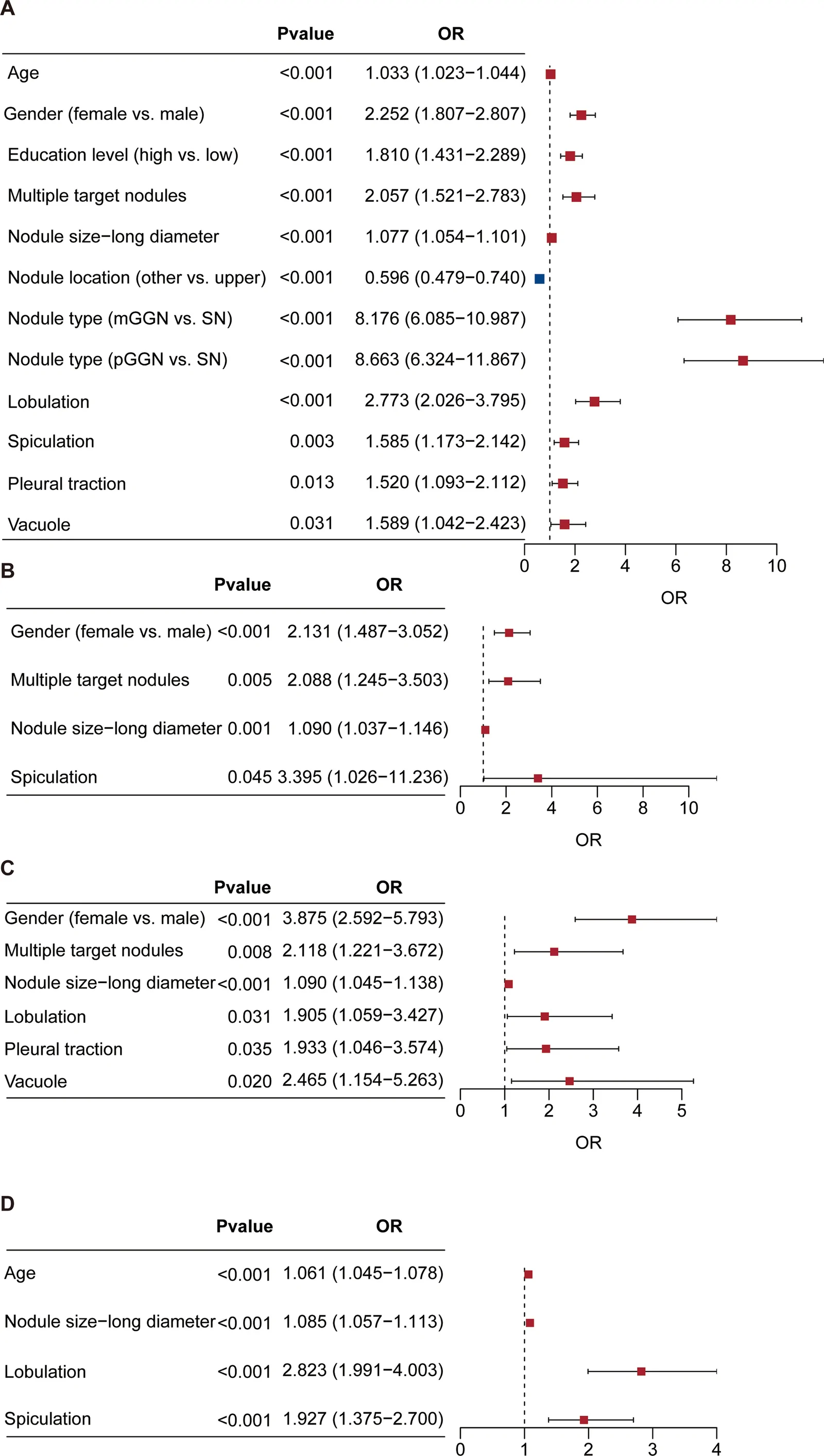
Risk factors associated with malignant nodules. (A), all the samples with outcomes. (B), pGGN samples. (C), mGGN samples. (D), SN samples. All the factors significantly associated with malignant nodules in the univariate analysis were subjected to multivariate factor binary logistic regression analysis via R software, version 4.0.0. *P-*value lower than 0.05 is significant. Forest plots were plotted with the “forestplot” (version 2.0.1) R package. The Hosmer-Lemeshow test was used to evaluate the goodness-of-fit of logistic regression models in Fig. 3A-D and shown in Supplementary Table 11. mGGN, mixed ground-glass nodule; OR, odds ratio; pGGN, pure ground-glass nodule; SN, solid nodule.

A total of 1692 (16.02%) patients with 1857 (13.94%) pulmonary nodules underwent surgical resection (including dozens of invasive biopsies) at the baseline from October 2018 to May 2021, after being assessed as high risk of lung cancer. As a result, a total of 1394 (13.20%) patients harboring 1493 malignant nodules and 298 (2.82%) patients harboring 364 benign nodules were confirmed through tissue pathological tests. To more accurately assess the differences between malignant and benign nodules or participants, we utilized follow-up data from October 2018 to September 2023. During the follow-up, 828 (7.8%) patients underwent surgical resection or invasive biopsy, with 729 patients (6.90%) diagnosed with malignant nodules and 99 patients (0.94%) with benign nodules. Additionally, 226 (2.14%) participants’ pulmonary nodules resolved and were classified as benign. We then grouped all confirmed malignant and benign participants, both at baseline and follow-up, for malignant-risk factor analysis (Table 1). All the pathological diagnoses were based on the 2015 World Health Organization (WHO) Histological Classification of Lung Cancer.^15^ The possible risk factors are listed in Supplementary Table 6. All factors significantly associated with malignant nodules in the univariate analysis (*P* < 0.05) were then subjected to multivariate analysis. The results revealed that age, female, education level (college or above), multiple target nodules, nodule size, nodule location (upper lobe *vs.* middle & lower lobe), nodule type (mGGN *vs.* SN, pGGN *vs.* SN), lobulation, spiculation sign, pleural retraction, vacuole of nodules were the independent risk factors closely associated with lung cancer (*P* < 0.05) (Fig. 3A and Supplementary Table 11). If adenocarcinoma *in situ* (AIS) is categorized as benign nodules, only the vacuole factor is excluded from the risk factors associated with malignancy (Supplementary Fig. 3and Supplementary Table 11).

Compared to benign nodules, malignant nodules were more commonly found in females (64.15% in female*vs.* 35.85% in male, *P* < 0.001) and in older patients (median age 55 *vs.* 52, *P* < 0.001) (Table 1). A larger proportion of participants with malignant nodules were non-smokers (78.05% nonsmokers *vs.* 21.95% smokers, *P* < 0.001), particularly among females, with 98.46% of female patients with malignant nodules being non-smokers, compared to 58.48% in males. The majority of male smokers (79.78%) and female nonsmokers (65.25%) with malignant nodules were over 50 years old (Supplementary Fig. 2).

Although the majority of nodules were solitary in the total population (81.24%), multiple nodules were more commonly observed in patients with malignant nodules than in those with benign ones (22.89% *vs.* 12.36%, *P* < 0.001) (Table 1). The long and short diameters of malignant nodules (10 mm *vs.* 9 mm, *P* < 0.001) were also larger than those of benign nodules. Most malignant nodules were located in the upper lobe of the lung (60.35%, *n* = 1394/2310), whereas more benign nodules were found in the middle and lower lobe (54.22%, *n* = 418/771) (*P* < 0.001) (Supplementary Table 3).

### 3.7 Pathological and morphological features of malignant nodules detected by CT

An impressive 97.32% (*n* = 2248/2310) of pathologically confirmed malignant nodules were diagnosed as lung adenocarcinoma, including 48.96% (*n* = 1131/2248) of invasive adenocarcinoma (IA), 33.85% (*n* = 782/2248) of minimally invasive adenocarcinoma (MIA), and 10.69% (*n* = 247/2248) of AIS (Table 2). According to the AJCC staging, 90.35% (2087/2310) of malignant nodules were at very early stages (0-I), with 10.74% (*n* = 248/2087) at stage 0, 40.9% (*n* = 933/2087) at stage IA1, 27.14% (*n* = 627/2087) at stage IA2, and 6.93% (*n* = 160/2087) at stage IA3. Most benign nodules were classified as inflammation (43.81%, *n* = 223/509), followed by atypical adenomatous hyperplasia (AAH) (12.57%), tuberculosis (9.43%), and hamartoma (8.06%) (Table 2).

**Table 2:** Pathological subtypes and AJCC staging of pathologically confirmed pulmonary nodules.

Characteristics	At baseline	At follow-up⁎	Total
Pathological subtypes-malignant, No. (%)			
Adenocarcinoma	1450 (97.12)	798 (97.67)	2248 (97.32)
AIS	136 (9.11)	111 (13.59)	247 (10.69)
MIA	448 (30.01)	334 (40.88)	782 (33.85)
IA	798 (53.45)	333 (40.76)	1131 (48.96)
Other adenocarcinoma	68 (4.55)	20 (2.45)	88 (3.81)
Squamous carcinoma	24 (1.61)	5 (0.61)	29 (1.26)
Small cell carcinoma	2 (0.13)	1 (0.12)	3 (0.13)
Large cell carcinoma	1 (0.07)	1 (0.12)	2 (0.09)
Malignant-other	16 (1.07)	12 (1.47)	28 (1.21)
Total	1493 (100.00)	817 (100.00)	2310 (100.00)
Pathological subtypes-bengin, No. (%)			
AAH	34 (9.34)	30 (20.69)	64 (12.57)
Inflammation	164 (45.05)	59 (40.69)	223 (43.81)
Tuberculosis	42 (11.54)	6 (4.14)	48 (9.43)
Hamartoma	33 (9.07)	8 (5.52)	41 (8.06)
Sclerosing pneumocytoma	12 (3.30)	5 (3.45)	17 (3.34)
Fungal infection	6 (1.65)	1 (0.69)	7 (1.38)
Benign-other	73 (20.06)	36 (24.83)	109 (21.41)
Total	364 (100.00)	145 (100.00)	509 (100.00)
AJCC stage, n (%)			
0	137 (9.18)	111 (13.59)	248 (10.74)
IA1	608 (40.72)	325 (39.78)	933 (40.39)
IA2	441 (29.54)	186 (22.77)	627 (27.14)
IA3	124 (8.31)	36 (4.41)	160 (6.93)
IB	92 (6.16)	27 (3.30)	119 (5.15)
IIA	2 (0.13)	1 (0.12)	3 (0.13)
IIB	19 (1.27)	7 (0.86)	26 (1.13)
IIIA	24 (1.61)	7 (0.86)	31 (1.34)
IIIB	1 (0.07)	2 (0.24)	3 (0.13)
IVA	12 (0.80)	5 (0.61)	17 (0.74)
IVB	4 (0.27)	0 (0.00)	4 (0.17)
Unknown	29 (1.94)	110 (13.46)	139 (6.02)
Total	1493 (100.00)	817 (100.00)	2310 (100.00)

⁎ The follow up period is from October 2018 to September 2023. Abbreviations: AAH, atypical adenomatous hyperplasia; AIS, adenocarcinoma in situ; AJCC, American Joint Committee on Cancer; IA, invasive adenocarcinoma; MIA, minimally invasive adenocarcinoma.

Malignant nodules were predominantly found in mGGNs (42.68%) and pGGNs (36.45%), while benign nodules were more common in SNs (50.84%) (Supplementary Table 3). Malignant SNs were mostly found in larger sizes, with 31.40% in the 20–30 mm range and 51.61% in the 10–20 mm range. Malignant mGGNs were mainly in the 10–20 mm size category (46.91%), whereas most malignant pGGNs were first detected as very small nodules, with 63.49% at 5–10 mm (Supplementary Table 7). Nodules that disappeared during follow-up were frequently observed in the 5–10 mm range (79.39%, *n* = 208/262) and SNs (40.84%, *n* = 107/262) (Supplementary Table 3).

Further analysis using multivariate regression on risk factors associated with lung cancer across the three nodule types revealed that female and multiple target nodules were common risk factors for GGNs (Supplementary Table 8), while lobulation and vacuole signs and pleural retraction were specific for mGGN and spiculation sign was specific for pGGNs (Fig. 3B and C, Supplementary Table 9 and 11). Nodule size, age and signs of spiculation and lobulation were risk factors for SNs (Fig. 3D, Supplementary Table 9 and 11).

### 3.8 Incorrect identification of benign and malignant nodules

For nodules of different sizes at baseline and during follow-up, we observed that large nodules (20–30 mm) had the highest surgery rate of 70.72% (*n* = 372/526), with a proportion of benign nodules (here *i.e.* the rate of pathologically confirmed benign cases among all the surgical cases) of 15.05% (*n* = 56/372). Small nodules (5–10 mm) accounted for the largest proportion of all nodules yet had the lowest surgery rate at 12.66% (*n* = 1320/10,428), but a high proportion of benign nodules of 20.15% (*n* = 266/1320) (Supplementary Table 10). Among GGNs and SNs, pGGNs displayed a medium surgery rate at 19.02% (*n* = 975/5127) and a proportion of benign nodules of 13.64% (*n* = 133/975). In contrast, mGGNs had the highest surgery rate of 33.62% (*n* = 1077/3203) and the lowest benign nodules proportion of 8.45% (91/1077). SNs had a low surgery rate of 15.33% (*n* = 766/4997) but the highest benign proportion of 37.21% (*n* = 285/766) (Supplementary Table 10). In this study, most of definitively pathological diagnoses were down via surgical resection (around 90%). Such a high benign proportion indicates there would be certain overtreatment (*i.e.* benign nodules were surgical resection) in clinical practice. Overtreatment can also refer to the surgical resection of true malignant lesions at very early stages, such as AIS lesions, which were not growing and/or were indolent, meaning they were unlikely to progress or cause symptoms or death over a long follow-up period. Our data revealed that approximately 10% of AIS and 33% of MIA lesions were pathologically diagnosed in the resected pulmonary nodules (Table 2), based on the lung cancer guidelines issued prior to the 2021 WHO classification.^15^^,^^20^ These findings suggest that distinguishing between benign and malignant small, solid, or indolent nodules is challenging, making it difficult to reduce overtreatment.

On the other hand, according to the Chinese Expert Consensus for lung cancer screening (2018 edition) ,^14^ 30.05% (*n* = 3173/10,560) of participants in this cohort met the high-risk criteria (age ≥ 40 years and ≥ 20 pack-years of tobacco smoking history, quitting smoking within the last 15 years, a history of high-risk occupational exposure (*e.g.*, asbestos, plating, uranium, radon), or a history of COPD, diffuse pulmonary fibrosis, pulmonary tuberculosis, malignant tumors, personal history of malignancy, or a family history of lung cancer). Of these, 21.78% (*n* = 691/3173) underwent surgery at baseline, leading to a lung cancer detection rate of 17.93% (*n* = 569/3173). For the remaining 69.95% of the population classified as non-high-risk, the surgery rate was 24.76% (*n* = 1829/7387), and the lung cancer detection rate was 21.04% (*n* = 1554/7837). As a result, the rate that the lung cancer cases undetected in the non-high-risk group compared to all lung cancer cases in this cohort was 73.20% (*n* = 1554/2123). Similar findings were observed based on NCCN guidelines (Supplementary Fig. 4). These results suggest that a significant number of lung cancer cases may be missed in non-high-risk populations as currently defined, highlighting the need for revisions to these criteria.

## 4 Discussion

Like most cancer types, the risk of developing lung cancer increases with age. As of 2020 in the United States, 70% of lung cancer diagnoses occurred in individuals aged 65 or older, while fewer than 5% were in those under 45 years old.^21^ In China, data up to 2024 showed that the highest incidence of lung cancer was at age over 60.^22^ However, since the introduction of LDCT for lung cancer screening in 2015, more pulmonary nodules have been detected in younger populations.^23^ In this large multicenter study of pulmonary nodules in China, we found that nearly 50% of lung nodules and lung cancers were diagnosed between the ages of 45–62 and 47–63, respectively, with 25% occurring in individuals aged 20–46. Our previous meta-analysis of lung cancer screening studies, encompassing 117,586 participants from 26 studies worldwide, revealed that the proportion of stage I lung cancer cases detected was higher in individuals aged 40–45 (approximately 80%) compared to those aged 50–55 (around 70%−60%), suggesting that a significant number of early-stage lung cancer cases already exist in individuals aged 40–50.^24^ Our data supported that lung cancer screening age starting from age 40 as recommended in the Chinese Expert Consensus for lung cancer screening.^14^ The growing incidence of pulmonary nodules in young individuals may be due to the increased adoption of LDCT for lung cancer screening, as well as a growing awareness of routine health check-ups, particularly among individuals with higher education levels. This aligns with our observation that malignant nodules were more frequently detected in the participants with high education levels (college and above). Although the reason behind this is still unclear, it could be due to increasing awareness or higher rates of screening in young and higher education people (or potential confounding factors in self-report in survey). Additionally, worsening air pollution over the past decades, for example, increased 11.2% of PM 2.5 concentration from 1990 to 2015 could be associated with increasing lung cancer incidence and deaths and contributed to this trend.^25^ These findings emphasize the importance of early screening and diagnosis of lung cancer in younger populations.

We also observed a higher prevalence of females with lung nodules compared to males under 50 years old in our cohort and lung cancers. In China, the age-standardized incidence rates of lung cancer increased by about 0.8% annually from 2000 to 2016, with a stable trend among men but a 2.1% year-on-year increase among women. Notably, the increase in lung cancer incidence among women in the < 40, 40–49, and 50–59 age groups were approximately 5.1%, 4.2% and 3.8% per year, respectively.^17^

In our study, 98.46% of female lung cancer patients were never-smokers, consistent with previous reports that show a high proportion of non-smokers among female with lung cancers in China, ranging from 50% to 94%.^8^^,^^10^^,^^17^ While smoking is a as well-established risk factor for lung cancer - accounting for 80% of lung cancer burden in male and >50% in female worldwide^26^- East Asian women with lung cancer have significantly lower smoking rates compared to their Caucasian counterparts. Studies have reported smoking prevalence among female lung cancer patients at 9.9% in The Republic of Korea, 17%−25.6% in Japan, and only 5.2% in China, whereas it ranges from 53% to 91% in Caucasian female patients.^10^^,^^21^ High proportion of non-smokers, young people and females in our cohort may explain the high rate of lung adenocarcinoma diagnosed in this study compared with other cohorts (72.5% and 53.1%).^18^^,^^27^ The underlying reasons for the increasing incidence of lung cancer in female non-smokers remain unclear. However, several studies reported that cooking oil fume exposure, second-hand smoking exposure, familial history of cancers, and gene mutations may be the significant risk factors for lung cancer in female non-smokers.^28^, ^29^, ^30^ In our cohort, 70.73% of females reported a history of second-hand smoke exposure, a rate comparable to 71.51% of males. Additionally, females had a higher prevalence of family history of cancer (23.85% *vs.* 21.26%) and lung cancer in first-degree relatives (14.40% *vs.* 12.40%) compared to males. These findings suggest that genetic predisposition and environmental exposures may contribute to the higher prevalence of pulmonary nodules and lung cancer among non-smoking females in China. Given these risks, it is crucial for women under 50, regardless of smoking history, to prioritize pulmonary nodule screening as part of routine health check-ups.

GGNs are a distinct type of pulmonary nodule type with notable difference in prevalence between Asia and Western regions.^31^ Patients with GGNs are typically female, nonsmokers, of Asian descent and young,^32^ consistent with our study findings. Our large-scale investigation further showed a significant association between pGGNs and a higher proportion of participants with a family history of lung cancer in first-degree relatives - particularly among sisters - compared to mGGNs and SNs. This suggests a potential genetic predisposition specific to the Chinese population or individuals of Asian descent, warranting further research.

Accumulated evidence have linked GGNs to lung cancer development, as slow-growing or stable GGNs often indicate early-stage lung cancer or preinvasive lesions.^33^ However, malignant GGN tend to exhibit “indolent” growth, few distant metastases, and favorable prognosis, with a 5-year survival rate of up to 100% after surgical resection.^34^ These findings emphasize the need for proper management of GGNs to identify their degree of invasiveness accurately, aiding clinical decision-making after LDCT screening.

Despite following the high-risk criteria outlined in Chinese guidelines for early lung cancer diagnosis and screening,^14^^,^^35^^,^^36^ a significant proportion of lung cancer patients (21.04%) emerged from individuals classified as "non-high-risk," in addition to the 17.93% identified within the high-risk group. This suggests that the development of pulmonary nodules and lung cancer in the Chinese population is influenced by a complex interplay of factors beyond the current high-risk definitions. Therefore, there is a pressing need to refine and expand the criteria for identifying high-risk individuals to improve early detection and intervention strategies.

However, there are several limitations to consider. First, the use of competitive enrollment may have led to an imbalance in participant numbers across research centers although the same study protocol and inclusion and exclusion criteria are used among all the centers. Second, certain epidemiological factors, such as occupational exposure and disease history, were self-reported, which may introduce misclassification bias. Additionally, while our study provides valuable insights into the clinical and imaging characteristics of indolent and active malignant pulmonary nodules, particularly GGNs, the completion of the three-year follow-up will allow for a more robust analysis with a significantly larger sample size. Lastly, as this is not a randomized controlled trial, further investigation is needed to determine whether including younger female never-smokers in lung cancer screening programs would lead to a reduction in lung cancer mortality or result in overtreatment.

In summary, our study is one of the largest prospective investigations documenting the demographic, clinical, and CT imaging characteristics of pulmonary nodule patients from 23 hospitals across 16 provinces in China. Our findings suggest that lung cancer screening should consider additional risk factors beyond smoking and occupational exposure, including younger female demographics, GGNs, multiple nodules, cooking habits, living environments, and broader environmental influences. These insights have important implications for refining risk assessment models and improving early detection strategies, both in China and globally.

## Ethics statement

This multicenter, prospective, observational cohort study (NCT03651986) was conducted in compliance with the principles of the Declaration of Helsinki and approved by the Ethics Committee of 23 participating hospitals in China^13^ (Supplementary Table 1). Written informed consent was obtained from all the participants.

## Funding

This work was supported by the R&D Program of Guangzhou National Laboratory Grant (grant numbers: 2023ZD0519700, GZNL2023A02007 and SRPG22–017); the Noncommunicable Chronic Diseases-National Science and Technology Major Project (grant number: 2024ZD0529501/2024ZD0529500); National Key Technology Special Projects (2024ZD0520004); China National Science Foundation (grant numbers: 82022048; 82373121); the Science and Technology Planning Project of Guangzhou (grant numbers: 202206080013); the National Key Research & Development Programme (grant number: 2022YFC2505100); Scheme of Guangzhou Economic and Technological Development District for Leading Talents in Innovation and Entrepreneurship (grant number: 2017-L152); Scheme of Guangzhou for Leading Team in Innovation (grant number: 201909010010); Yunnan Provincial People’s Hospital Cooperation Project (grant number: 202201AY070001–224); The Science and Technology Project of Gansu Province (grant number: 20JR10FA666); Wu-Jieping Medical Foundation (grant number: 320.6750.2020–05–1); National Science Foundation of China (grant number: NSFC81925001); Shenzhen Science and Technology Program (grant number: JCYJ20220530152414032); The Central Government Guides Local Science and Technology Development Fund Project (grant number: 2022ZY0127); Medical Master Project of Xingliao Talent Plan (grant number: YXMJ-LJ-07); Sanqin Special Supporting Program Innovation and Entrepreneurship Team: Precise Diagnostic and Therapeutic Regimes & Procedure Standardized Management in Lung Cancer; Interventional Oncology at Johnson and Johnson.

## Declaration of competing interest

Minhua Peng, Shuangxiu Wu, Liuhong Zeng, Lin Teng, Xiangcheng Qiu, Bo Wang, Jinsheng Tao, Meng Yang, Junjie Zhao and Jianbing Fan are current employees of AnchorDx Medical or AnchorDx. Silvia S. Chen is current employee of Johnson and Johnson. All other authors declare that they have no known competing financial interests or personal relationships that could have appeared to influence the work reported in this paper. Patients or the public were not involved in the design, conduct, reporting, or dissemination of this research.

## CRediT authorship contribution statement

**Wenhua Liang:** Conceptualization, Data curation, Formal analysis, Investigation, Methodology, Resources, Funding acquisition. **Yongzhao Zhou:** Data curation, Investigation, Resources. **Chengping Hu:** Data curation, Investigation, Resources. **Wei Xiao:** Data curation, Investigation, Resources. **Jian Zhang:** Data curation, Investigation, Resources. **Min Li:** Data curation, Investigation, Resources. **Wei Wang:** Data curation, Investigation, Resources. **Xianwei Ye:** Data curation, Investigation, Resources. **Xiangyan Zhang:** Data curation, Investigation, Resources. **Ning Chang:** Data curation, Investigation, Resources. **Chunmei Yun:** Data curation, Investigation, Resources. **Dejun Sun:** Data curation, Investigation, Resources. **Yunhui Zhang:** Data curation, Investigation, Resources. **Zheng Qin:** Data curation, Investigation, Resources. **Wei Wang:** Data curation, Investigation, Resources. **Yong Zhang:** Data curation, Investigation, Resources. **Xin Zhang:** Data curation, Investigation, Resources. **Yi Xiang:** Data curation, Investigation, Resources. **Jieming Qu:** Data curation, Investigation, Resources. **Wei Zhang:** Data curation, Investigation, Resources. **Longhua Sun:** Data curation, Investigation, Resources. **Yang Hu:** Data curation, Investigation, Resources. **Jinfu Xu:** Data curation, Investigation, Resources. **Di Wu:** Data curation, Investigation, Resources. **Jianping Zhao:** Data curation, Investigation, Resources. **Xiaoju Zhang:** Data curation, Investigation, Resources. **Hairong Bao:** Data curation, Investigation, Resources. **Xiaoju Liu:** Data curation, Investigation, Resources. **Yubiao Guo:** Data curation, Investigation, Resources. **Zhanqing Li:** Data curation, Investigation, Resources. **Qichuan Zhang:** Data curation, Investigation, Resources. **Weiyi Zhu:** Data curation, Investigation, Resources. **Lan Yang:** Data curation, Investigation, Resources. **Xiaomin Dang:** Data curation, Investigation, Resources. **Baohui Han:** Data curation, Investigation, Resources. **Zhaohui Tong:** Data curation, Investigation, Resources. **Feng Wang:** Investigation. **Liping Liu:** Data curation, Investigation, Resources. **Minhua Peng:** Writing – original draft, Formal analysis. **Shuangxiu Wu:** Writing – original draft, Formal analysis, Project administration. **Liuhong Zeng:** Formal analysis. **Lin Teng:** Project administration. **Xiangcheng Qiu:** Formal analysis, Project administration. **Bo Wang:** Formal analysis, Project administration. **Jinsheng Tao:** Formal analysis. **Meng Yang:** Formal analysis. **Junjie Zhao:** Formal analysis. **Silvia S. Chen:** Formal analysis. **Jianbing Fan:** Conceptualization, Funding acquisition, Formal analysis, Methodology. **Weimin Li:** Funding acquisition, Investigation, Resources. **Dan Liu:** Funding acquisition, Investigation, Resources. **Jianxing He:** Funding acquisition, Data curation, Investigation, Resources. **Nanshan Zhong:** Conceptualization, Funding acquisition, Methodology.

## Appendix Supplementary materials

Image, application 1

Image, application 2
