# Supplementary material for: Epidemiological characteristics and risk factors of lung cancer in a population with incidental pulmonary nodules in China: a prospective multicenter study of 10,560 cases
Source: J Natl Cancer Cent. 2026 Apr 16;6(4):379–90. doi: 10.1016/j.jncc.2026.03.003 (PMC13494645; doi:10.1016/j.jncc.2026.03.003)
Supplement: Supplementary file 1 [file mmc1.pdf]

Supplementary materials

Epidemiological characteristics and risk factors of lung cancer in a population with incidental pulmonary nodules in China: a prospective multicenter study of 10,560 cases

Wenhua Liang, Yongzhao Zhou, Chengping Hu, Wei Xiao, Jian Zhang, Min Li, Wei Wang, Xianwei Ye, Xiangyan Zhang, Ning Chang, Chunmei Yun, Dejun Sun, Yunhui Zhang, Zheng Qin, Wei Wang,Yong Zhang, Xin Zhang, Yi Xiang, Jieming Qu, Wei Zhang, Longhua Sun, Yang Hu, Jinfu Xu, Di Wu, Jianping Zhao, Xiaoju Zhang, Hairong Bao, Xiaoju Liu, Yubiao Guo, Zhanqing Li, Qichuan Zhang, Weiyi Zhu, Lan Yang, Xiaomin Dang, Baohui Han, Zhaohui Tong, Feng Wang, Liping Liu, Minhua Peng, Shuangxiu Wu, Liuhong Zeng, Lin Teng, Xiangcheng Qiu, Bo Wang, Jinsheng Tao, Meng Yang, Junjie Zhao, Silvia S. Chen, Jianbing Fan, Weimin Li, Dan Liu, Jianxing He, Nanshan Zhong

Supplementary methods

Discontinuing study interventions and patient withdrawal

Subjects may discontinue the study intervention prematurely for the following reasons:

(I) Subjects request to leave the trial i.e., patients withdraw informed consent and are free to leave the study at any time; (II) patient dies; (III) subjects are lost to follow-up; (IV) the researcher believes that patients do not follow instructions in protocol; (V) individuals who are judged inappropriate for the study by investigator. If a subject discontinues the intervention or chooses to discontinue the intervention prematurely, an early withdrawal visit will be completed without delay but no later than one week after discontinuation. The reason(s) for premature discontinuation will be documented accordingly. Every effort will be made to follow-up subjects who terminate prematurely to determine the final clinical outcome.

Sample size calculation

This study predicts that both the sensitivity and specificity of ctDNA methylation detection in the diagnosis of malignant pulmonary nodules, based on the pre-experimental results, will be

80%. At the tolerance of 0.05 (α = 0.05), at least 264 patients with benign pulmonary nodules and 264 patients with malignant pulmonary nodules should be included according to the calculation formula of diagnostic test sample size. Based on previous relevant studies, 3% of people with positive pulmonary nodules were diagnosed with lung cancer; considering a drop-out rate of 20%, the required sample size is *n* = 10,560. Thus, 10,560 participants will need to be enrolled for this trial.

Investigation about smoking history and exposure to second-hand smoke in the Questionnaire

The Questionnaire about smoking and secondhand smoke information were collected as in below table. The definition of a smoker is someone who currently smokes or has smoked in the past. Heavey smoker is defined as someone with a smoking history of 20 pack-years or more. The method for calculating pack-years of smoking is: the number of packs smoked per day (20 cigarettes per pack) multiplied by the number of years smoked. For secondhand smoke (i.e., passive smoking), its survey covers two situations: the living with cohabitants or the working environment. In both situations, exposing time should be equal to or more than 2 hours per day and then the number of years exposed is recorded. Those who have been exposed to secondhand smoke for equal to or more than 10 years are considered high-risk individuals. Pack-years of smoking and exposing years of secondhand smoke are used for calculate their risk of lung cancer.

Smoking history	Smoking state	Smoking amount	Smoking year	Secondhand smoke (living, ⩾2h/day)	Secondhand smoke (working, ⩾2h/day)	Exposing to Secondhand smoke year
Yes/No	current/ used /never	average cigarettes/day		Yes/No	Yes/No	

A

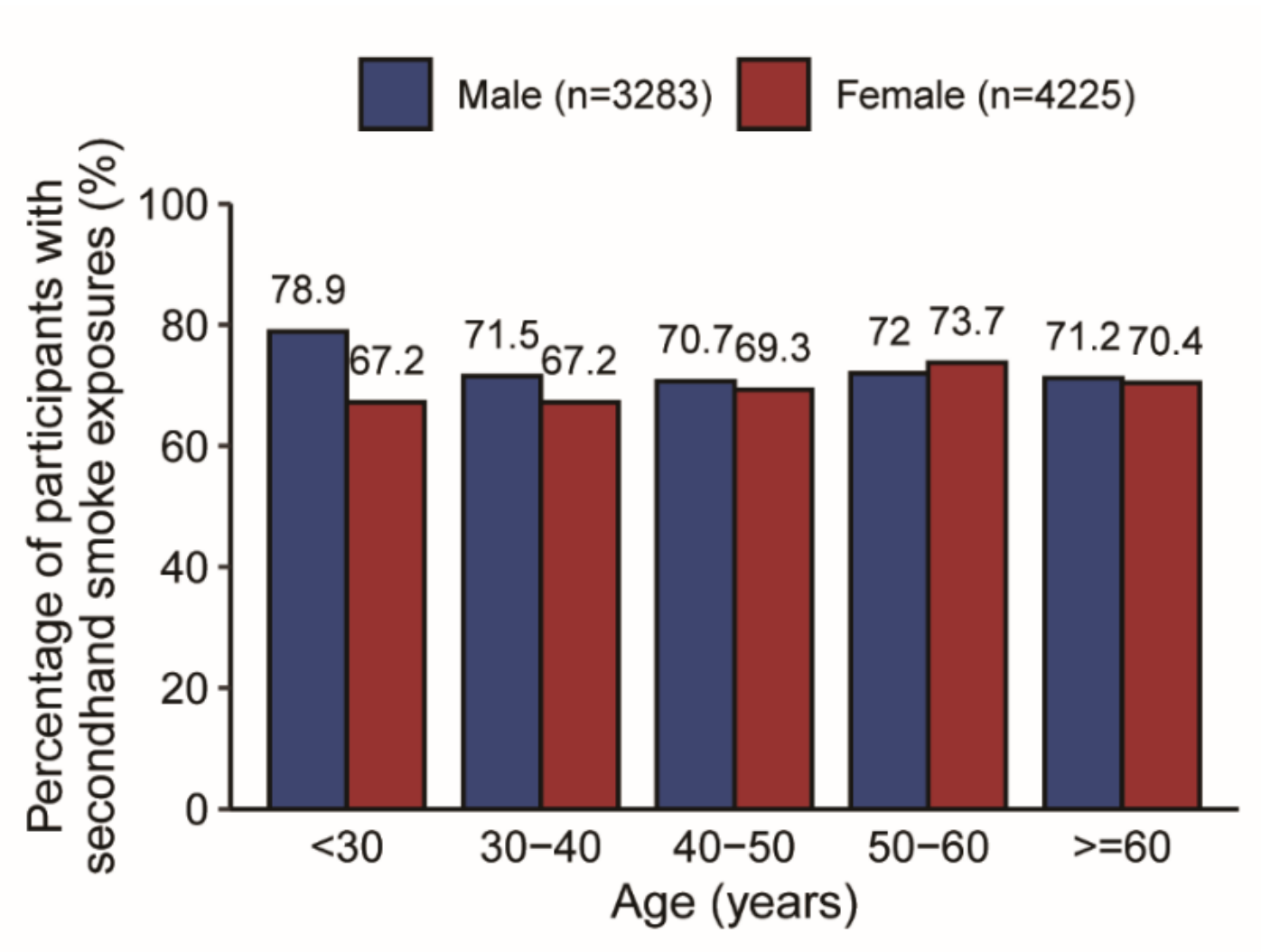

B

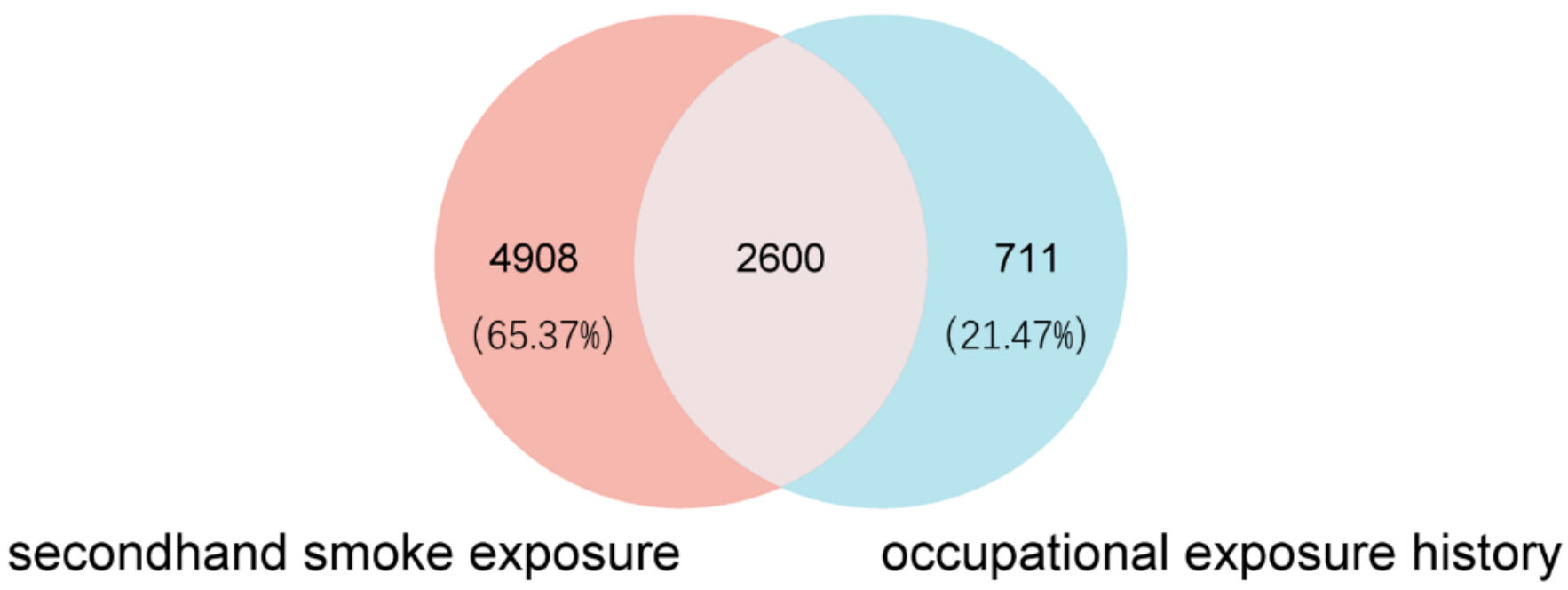

Supplementary Fig. 1. Distribution of the pulmonary-nodule population with secondhand-smoke history and occupational exposure history. (A) Distribution with secondhand-smoke history by gender and age. The numbers in the brackets are the numbers of males and females with secondhand-smoke history in the cohort. (B) Overlap population both with secondhand-smoke history and occupational exposure history.

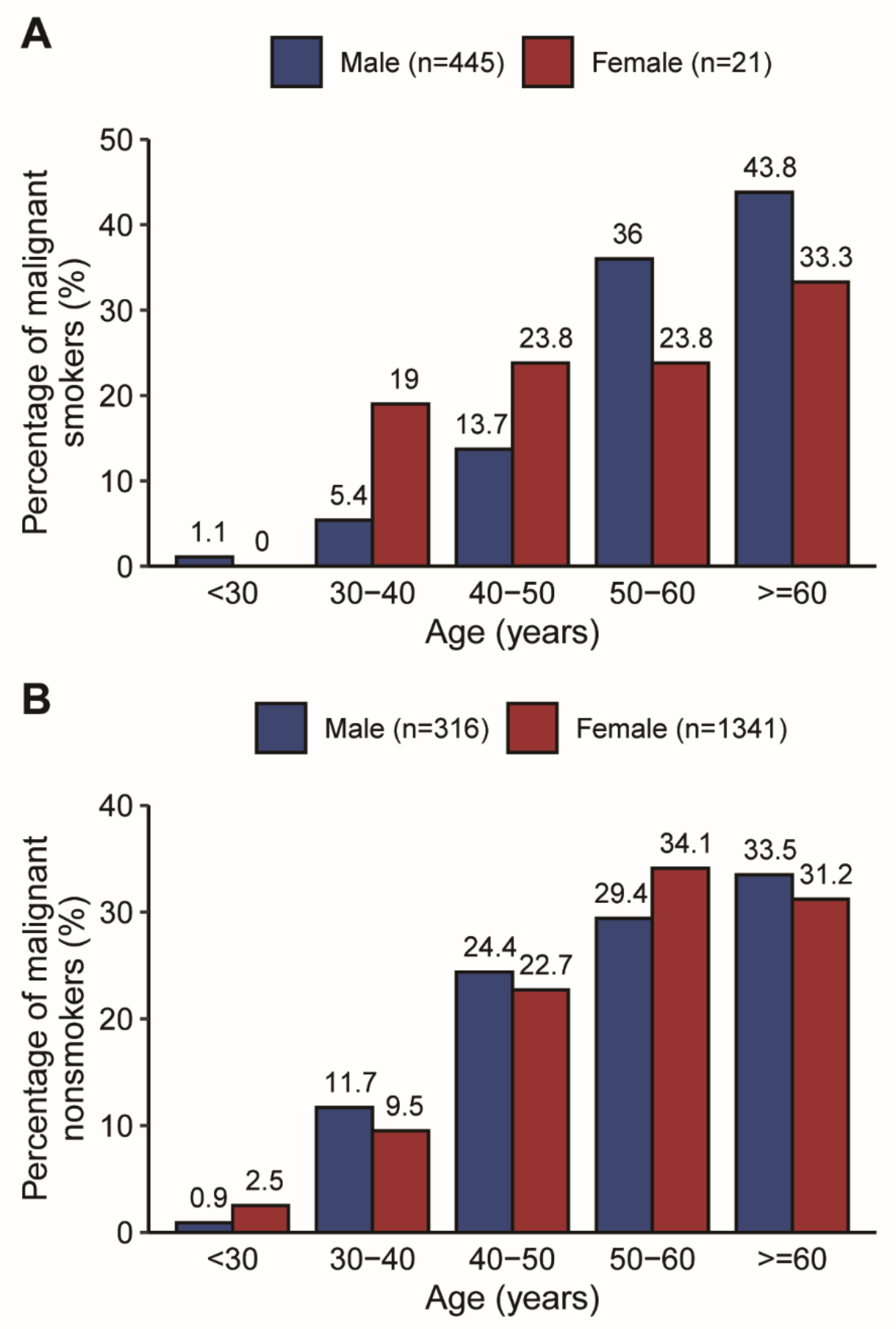

Supplementary Fig. 2. Distribution of malignant pulmonary-nodule population with smoking history by gender and age. (A) For the smokers. (B) For the non-smokers.

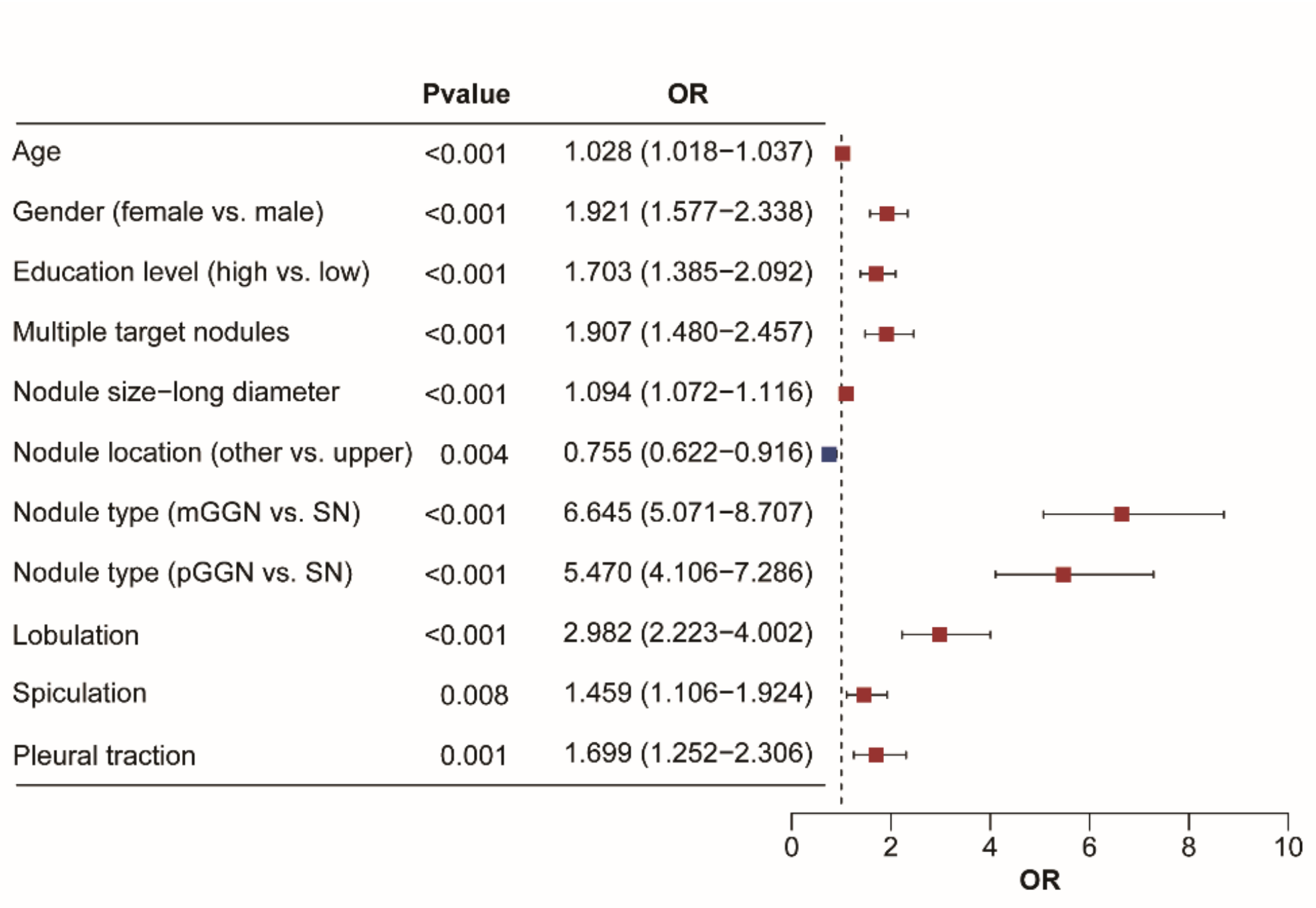

Supplementary Fig. 3. Risk factors associated malignant nodules which not included adenocarcinoma in situ. All the factors significantly associated with malignant nodules in the univariate analysis were subjected to multivariate factor binary logistic regression analysis via R software, version 4.0.0. *P* value lower than 0.05 is significance. Forest plots were plotted with the "forestplot" (version 2.0.1) R package. The Hosmer-Lemeshow test was used to evaluate the goodness-of-fit of logistic regression model in Supplementary Fig. 3 and shown in Supplementary Table 11. mGGN, mixed ground-glass nodule; pGGN, pure ground-glass nodule; SN, solid nodule.

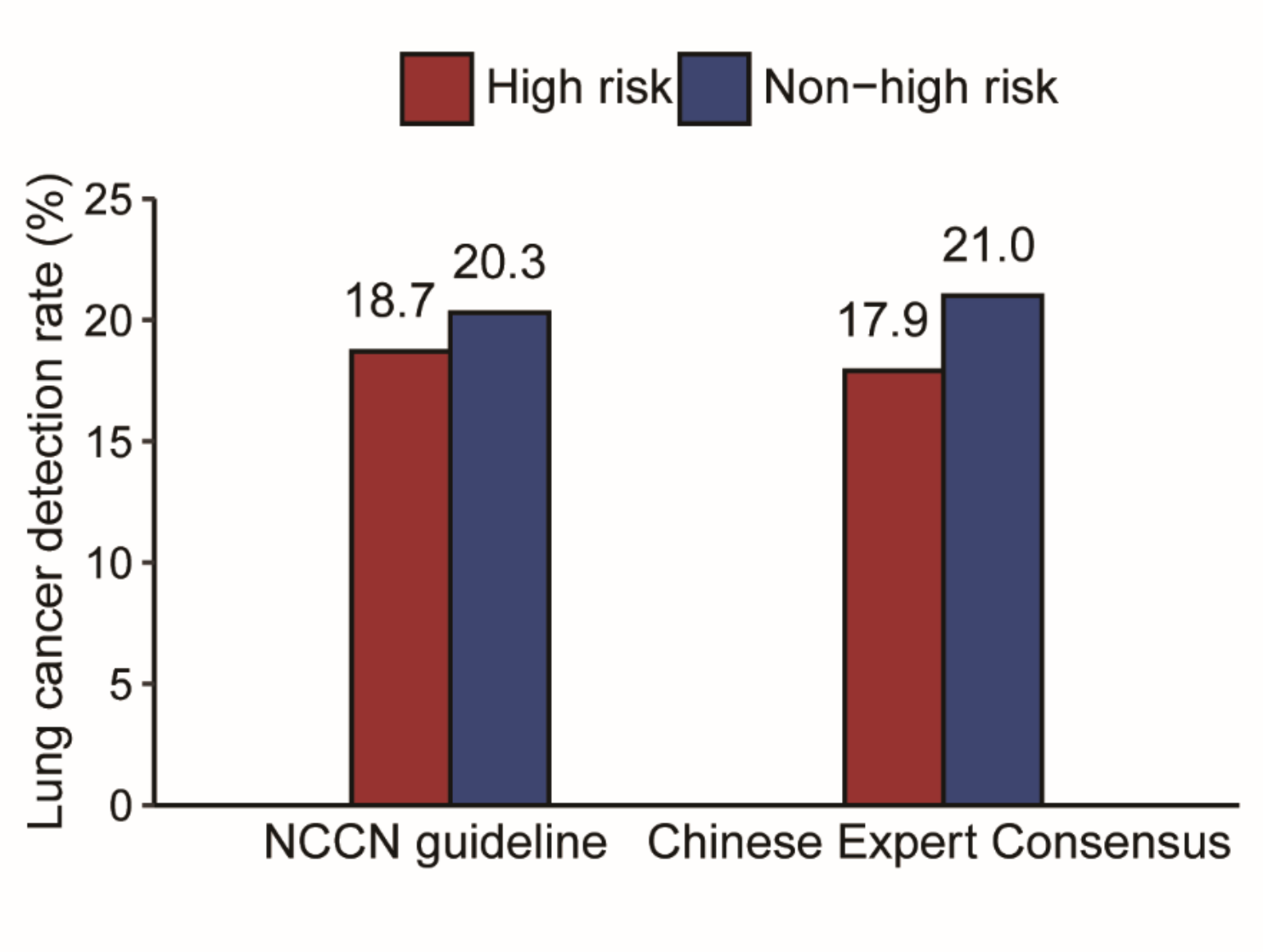

Supplementary Fig. 4. Lung cancer detection rate according to high-risk criteria in the Chinese Expert Consensus and NCCN guidelines for lung cancer early detection and careening.
